# Analysis of length of stay and treatment emergent complications in hospitalized myasthenia gravis patients with exacerbation

**DOI:** 10.1186/s12883-022-02922-9

**Published:** 2023-01-12

**Authors:** Taylor Ramsaroop, Deborah Gelinas, Seung Ah Kang, Raghav Govindarajan

**Affiliations:** 1 University of Missouri School of Medicine, Columbia, MO USA; 2 Department of Neurology, University of North Carolina - Chapel Hill, Chapel Hill, North Carolina USA; 3 Department of Neurology, University of Missouri Hospital, Columbia, MO USA

**Keywords:** Myasthenia gravis, Myasthenic crisis, Thymectomy, Outcome measure, Mechanical ventilation, Treatment, Hospitalization, Length of stay

## Abstract

**Introduction:**

AIMS

Myasthenia Gravis (MG) is an autoimmune neuromuscular disease in which patients suffer from recurrent exacerbation. There are insufficient data measuring the effects of the resources employed before and during acute exacerbation on subsequent disease outcomes. This study aims to identify factors which lead to lengthened hospital stay.

**Methods:**

This is a retrospective chart review of acute MG exacerbations requiring hospitalization. Exacerbations were identified using ICD-9/ICD-10 codes and considered the following variables: age and Myasthenia Gravis Foundation of America (MGFA) class at initial MG diagnosis, age and MGFA class at exacerbation, sex, thymectomy, cause of exacerbation, treatment regimen at time of exacerbation, inpatient treatment regimen, length of hospital stay (LOS), intubation, use of noninvasive ventilation, complications, and disposition.

**Results:**

Seventy patients with 141 hospitalizations were identified. Crisis management characterized by intubation and plasmapheresis positively correlated with LOS (both *p* < .001). Almost 1/5 hospitalizations required intubation. Previous thymectomy negatively correlated with LOS *(p* < .05). In contrast, male sex correlated with longer LOS (*p* < .05). One-third of hospital stays were followed by discharge to a post-acute care facility, 7% home with home health, and 1 hospitalization resulted in death.

**Discussion:**

Plasmapheresis, intubation, and male sex were associated with increased LOS in acute MG exacerbation. Intubation appears to be the strongest predictor of LOS. Those with previous thymectomy had shorter hospital stays. The role of thymectomy in the acute setting merits further analysis.

**Supplementary Information:**

The online version contains supplementary material available at 10.1186/s12883-022-02922-9.

## Introduction

Myasthenia Gravis (MG) is a chronic autoimmune neuromuscular disease with recurrent exacerbations [1]. Approximately 10% of patients with generalized MG have significant disease and recurrent exacerbations that require hospitalization [2, 3].

Patients with myasthenic crisis (worsening of myasthenic weakness requiring intubation or noninvasive ventilation) are at increased risk for infectious and vascular complications with the most common cause of death being multiorgan failure due to sepsis and respiratory failure despite maximal care [4, 5]. Risk factors for prolonged ventilation (> 15 days) include older age, higher baseline disease severity, and pneumonia [4].

A previous study concluded that refractory MG patients in England utilize more healthcare resources than non-refractory MG patients as determined by number of visits to healthcare providers and hospitalizations. Refractory was defined as having  ≥ 2 immunosuppressive therapies (IST) prescribed, > 3 treatment episodes of the same ISTs within 24 months (prescription was ≥ 90 days after the previous prescription for the same IST), or ≥ 1 IST prescribed plus ≥ 4 hospital treatments (plasmapheresis or intravenous immunoglobulins [IVIg]) ≥ 2 months apart within a year. All patients with MG not identified as refractory using this algorithm were considered non-refractory. Course of hospital stay, however, was not examined [6]. Another study on hospitalized MG patients in Thailand evaluated various predictors of poor outcome post-hospitalization and found hospital category (primary, secondary, tertiary, or private), presence of pneumonia and respiratory failure to be significant [7]. Poor outcomes were defined as a discharge status of “not improved” or dead. A more recent study (2017) concluded that pneumonia, mechanical ventilation, and septicemia were independent factors associated with poor outcomes in elderly hospitalized patients in Thailand [8]. Neither study discussed the factors which might have influenced length of stay (LOS).

This study aims to identify factors that determine length of hospital stay, associated hospital complications and discharge disposition among generalized MG patients with exacerbation.

## Methods

### Data acquisition

**Fig. 1:**
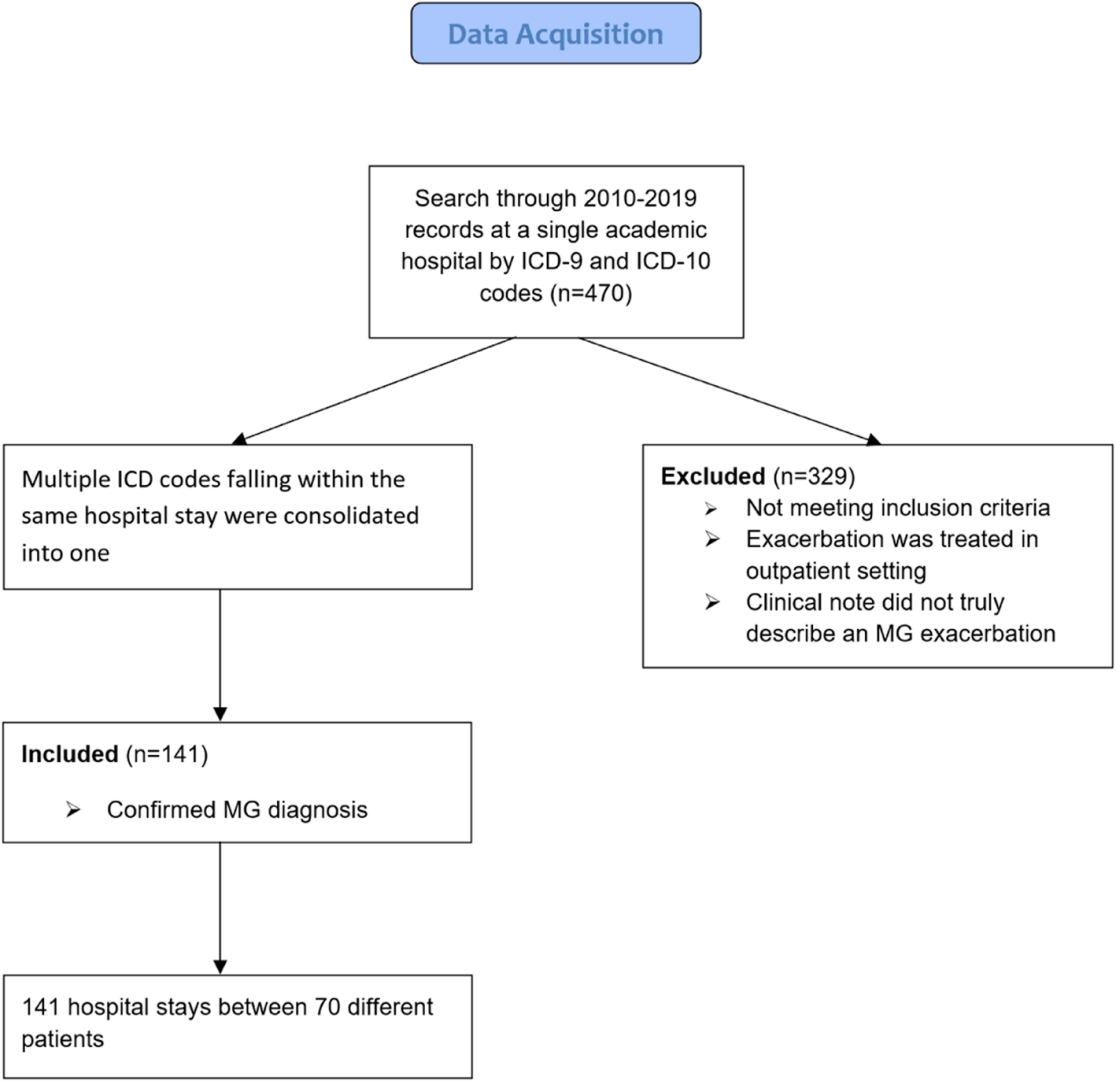
Data acquisition flow diagram illustrating case selection

This is a retrospective chart review of generalized MG exacerbations for which patients were hospitalized and treated at a single academic medical center between 2010 and 2019. This study was ethically conducted with approval by the local institutional review board. Exacerbations were identified using the codes for MG exacerbation from both ICD-9 and ICD-10 (358.01, G70.01). Using this method, a total of 470 exacerbations between 140 different patients were identified (Fig. 1).

### Inclusion criteria

A confirmed diagnosis of MG met at least one of the following criteria: (1) seropositivity (positive AChR-Ab, MuSK-Ab, or LRP4 assays in lab results or clinical documentation), (2) significant decrement (> 10%) on low frequency repetitive nerve stimulation or increased jitter with single fiber electromyogram, (3) long standing documented history of MG, referred from an outside MG center, or responsive to treatment but without electronic records of initial diagnostic tests [1]. Exacerbation was defined as generalized weakness and/or respiratory compromise that required inpatient monitoring and treatment.

### Exclusion criteria

A patient or exacerbation was excluded from this study if they met one or more of the following criteria: exacerbation was treated in outpatient setting, the clinical note did not describe a true exacerbation (routine follow-up, scheduled maintenance inpatient treatment, disease well managed and stable, no changes to treatment management required or considered), erroneous use of the ICD code (e.g. patient had MG but an unrelated condition was the primary ailment requiring treatment), or no confirmed diagnosis of MG. Multiple ICD codes falling within the same hospital stay were consolidated into one, such that one hospital stay represented one acute exacerbation. A total of 141 hospital stays for acute MG exacerbation between 70 different patients ultimately met inclusion criteria.

### Acute exacerbation

Acute exacerbation was defined as worsening of MG symptoms including dysphagia, ptosis, respiratory function, and decline in general physical function which necessitated either monitoring or treatment. Furthermore, MG or complications related to MG was the primary condition being managed for the duration of the hospital stay.

Causes of exacerbation were determined by explicit statement of a managing physician in the clinical notes and grouped into 8 categories: unknown, none identified due to first presentation of MG, medication-related (use of medications that are to be avoided or be used with caution in patients with MG [MUAC-MG]), medical non-compliance (medications not taken as prescribed due to inability to pay or for other reason as stated in patient chart), vaccine preventable infection (VPI), vaccine non-preventable infection (VNPI), procedure-related, and other (eg, social stress). VPI included pneumococcal pneumonia and seasonal influenza supported by laboratory findings. VNPI included unspecified respiratory (upper and lower tract), any other non-respiratory infections, and sepsis [1]. A single category was assigned to each exacerbation.

### Variables considered for length of stay

For each event the following variables were considered: age and MGFA class at time of initial diagnosis of MG, age and MGFA class at time of exacerbation, sex, race, antibody status, thymectomy status, cause of exacerbation, medications at time of exacerbation, length of hospital stay, location in hospital, intubation, use of non-invasive ventilation, treatment regimen, complications developed, and discharge disposition. These variables were identified through Electronic Medical Records (EMR) review.

### Complications of inpatient management of acute exacerbation

An event or medical problem was considered a treatment-related complication if a medication or therapy was explicitly said to have caused the patient significant impairment in the chart, required additional management beyond MG disease (eg, surgery), or affected the disease course and potentially length of stay (eg, catheter malfunction or catheter infection delaying IVIg treatment, non-tolerance of IVIg in form of nausea/vomiting which limited or delayed management).

### Statistical analysis

LOS was treated as a count variable. Previous literature and concerns for over-dispersion led to the use of negative-binomial regression [9–12]. We used a multivariable zero-truncated negative binomial model since LOS is a positive integer value. The relationship between LOS and other variables in the study was analyzed via both univariable and multivariable regression. The data reported here is that of the multivariable regression as this mode of analysis accounts for variables potentially dependent upon multiple independent variables [13]. All variables used can be found in Supplemental Table 1 . Coefficients and confidence intervals (CI) were exponentiated to determine the incidence rate ratios (IRR) and their confidence intervals. Significance level (α) was set at 5%. Due to multiple comparisons for MGFA class at initial MG diagnosis and for causes of exacerbation, we used Bonferroni correction to adjust *p*-values (α_new_). For MGFA class, α_new_ (α/6) was 0.00833. For exacerbation cause, α_new_ (α/8) was 0.00625. Results were considered significant if *p* < α_new_. All analyses were performed in RStudio Version 1.1.456, a freely accessible statistical software program.

## Results

### Patient population covariates

Age at time of MG diagnosis ranged from 12 to 92 years old with a mean of 53.44 years old (SD ± 19.85). The distribution of hospitalizations per MGFA class at initial MG diagnosis are described in Supplemental Table 2. Patient demographics including sex, race, seropositivity status, and thymectomy status are described in Table 1. It also includes the percentage of patients on a certain therapy as maintenance therapy prior to the exacerbation, including those on multiple medications. Patients not on any immunomodulatory treatment included some newly diagnosed patients.

**Table 1:** Patient population demographics and their maintenance treatment regimens at time of exacerbation

Population Demographic	Number of patients (%) (*n* = 70)
Sex (Male)	47%
Race – White	96%
Race—Black	4%
Seropositive Status^b^	63%
Thymectomy	21%
**Maintenance Treatment**	**Number (%) of maintenance regimens at time of exacerbation** **(*n* = 141)**
Steroid Sparing Agents^a^	26.2%
Maintenance IVIG	21.3%
Maintenance Plasmapheresis	7.1%
Prednisone	37.6%
Pyridostigmine	77.3%
Monoclonal Antibodies^†^	1.4%
> 1 Treatment	66%
None	16.3%

^a^Steroid Sparing Agents – Azathioprine (9.2%), Mycophenolate Mofetil (16.3%), Cyclosporine (0.7%). †Monoclonal Antibodies – Rituximab (0.7%), Eculizumab (0.7%)

^b^Positive AChR-Ab, MuSK-Ab, or LRP4 assays

### Events leading to exacerbation

**Fig. 2:**
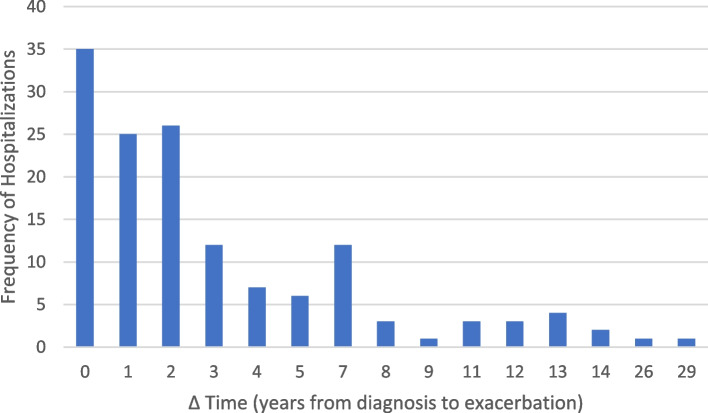
Frequency of Hospitalizations by Time from Diagnosis to Exacerbation

**Fig. 3:**
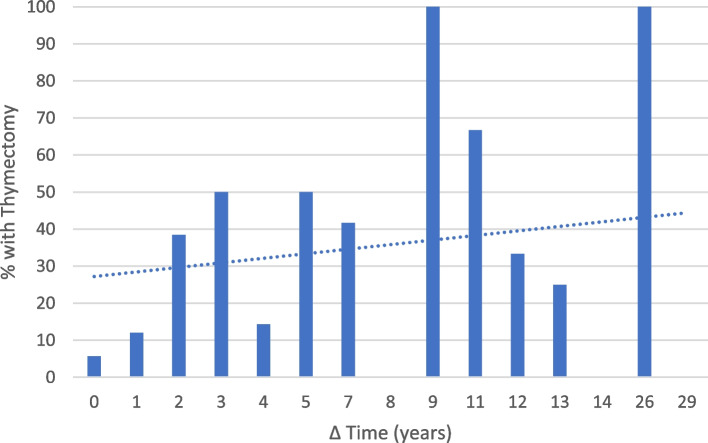
Percentage of Patients with Thymectomy per Number of Years from Diagnosis to Exacerbation

**Fig. 4:**
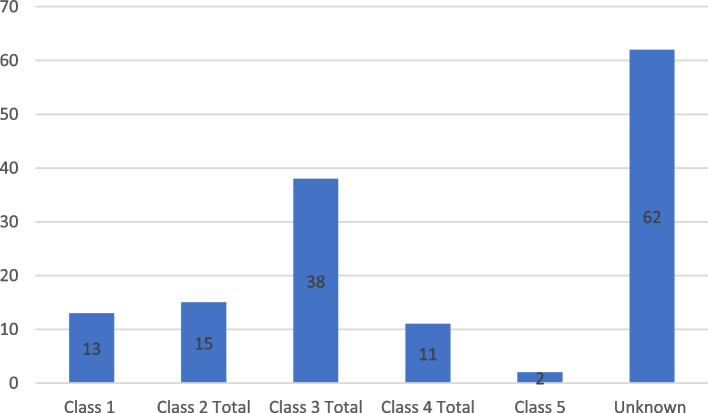
MGFA Class at Exacerbation. Class 2 Total includes: Class 2a (*n* = 4), Class 2b (*n* = 7), Class 2 not otherwise specified (*n* = 4). Class 3 Total includes: Class 3a (*n* = 14), Class 3b (*n* = 15), Class 3 not otherwise specified (*n* = 9). Class 4 Total includes: Class 4a (*n* = 3), Class 4b (*n* = 2), Class 4 not otherwise specified (*n* = 3)

For the exacerbations where cause could be identified VNPI and medication-related were the most common etiologies (Table 2). Average age at hospitalization (55.8 years ± 20.6) was very similar to the average age at initial MG diagnosis. Age at time of hospitalization ranged from 14 to 95 years old. The average length of time from initial MG diagnosis to time of hospitalization (Δtime or Δt (years) = Age at diagnosis-age at hospitalization) was 3.49 years, ranging from 0 to 29 years. Figure 2 illustrates a clear down-trend in frequency of exacerbations as time from diagnosis increases. Most hospitalizations (25%) occurred within the same year of initial diagnosis (Δt = 0 years). Excluding the outliers (Δt values where frequency of hospitalizations is less than 3/141), there was also the lowest rate of thymectomies within this period. There was an overall inverse relationship between rate of thymectomies and frequency of hospitalization (Fig. 3). Of the hospitalizations that occurred within the same year of initial diagnosis, 25% (*n* = 9) were in patients whose MG first presented as crisis and therefore did not carry a diagnosis of MG prior to hospitalization. When excluding those 9, patients at Δt = 0 still had the lowest thymectomy rate and greatest frequency of exacerbation (tied with Δt = 2). Of those where MGFA class at time of exacerbation was able to be determined, Class 3 was the most common (Fig. 4), whereas Class 2 was the most common at time of diagnosis.

**Table 2:** Number of hospitalizations per cause of exacerbation and management per event

Cause of Exacerbation	Number (%) (*n* = 141)
Unknown	42.55%
None Identified—New Diagnosis	6.4%
Medication-Related	12.76%
Non-Compliance	9.21%
Procedural	3.54%
Vaccine Non-preventable Infection (VPNPI)	12.76%
Vaccine Preventable Infection (VPI)	7.10%
Other	5.67%
**Exacerbation Management Per Event**	
IVIG	48.94%
Plasmapheresis	46.10%
IVIG and Plasmapheresis	7.09%
Intubations	17.73%
Use of Non-Invasive Ventilation	42.55%
Tracheostomy	7.1%
PEG^a^	17.7%

^a^Percutaneous Endoscopic Gastrostomy.

### Acute exacerbation treatment

ICU admission comprised 5% of the hospitalizations (Supplemental Table 3). There were 10 intubated and 15 non-intubated patients who required parenteral nutrition. Ten intubated patients required tracheostomies. One patient left prematurely against medical advice. IVIg and plasmapheresis were used at almost identical rates (Table 2). There were 10 exacerbations treated with both plasmapheresis and IVIg which occurred in 10 unique individuals (Table 3).

**Table 3:** Mean length of stay for type of exacerbation management and distribution of individuals undergoing each management type

Exacerbation Management Per Event (*n* = 141)	Mean Length of Stay in Days (SD)	Number of Unique Individuals (*n* = 70)
IVIG	6.38 (7.0)	43
No IVIG	10.69 (12.0)	27
Plasmapheresis	13.2 (12.8)	39
No Plasmapheresis	4.63 (4.1)	31
IVIG and Plasmapheresis	17.9 (10.52)	10
No IVIG or Plasmapheresis	4.14 (4.0)	13
Intubation	21.64 (17.0)	21
No Intubation	5.77 (4.3)	49
ICU	16.43 (5.4)	7
Non-ICU	8.17 (10.12)	63
Total	8.58 (10.1)	70

### Length of hospital stay

Mean length of hospital stay was 8.58 days (SD ± 10.1 days) and median was 6 days. Most hospitalizations were under 25 days (Supplemental Fig. 1). The average LOS for the 10 treated with both plasmapheresis and IVIg was longer than that of IVIg and plasmapheresis alone (Table 3).

## Factors affecting length of stay

### Factors associated with increased LOS

Intubation and treatment with plasmapheresis were strongly associated with increased length of hospital stay (*p* < 0.001, Supplemental Figure 2). Intubated patients had a much longer average LOS compared to non-intubated patients. The range for LOS in intubated and non-intubated patients were 5 to 92 and 1 to 30, respectively. Intubated and non-intubated patients had similar average ages at time of initial MG diagnosis (59.6 and 52.4 years respectively) and the most common MGFA class at diagnosis amongst both populations was Class 2.

**Fig. 5:**
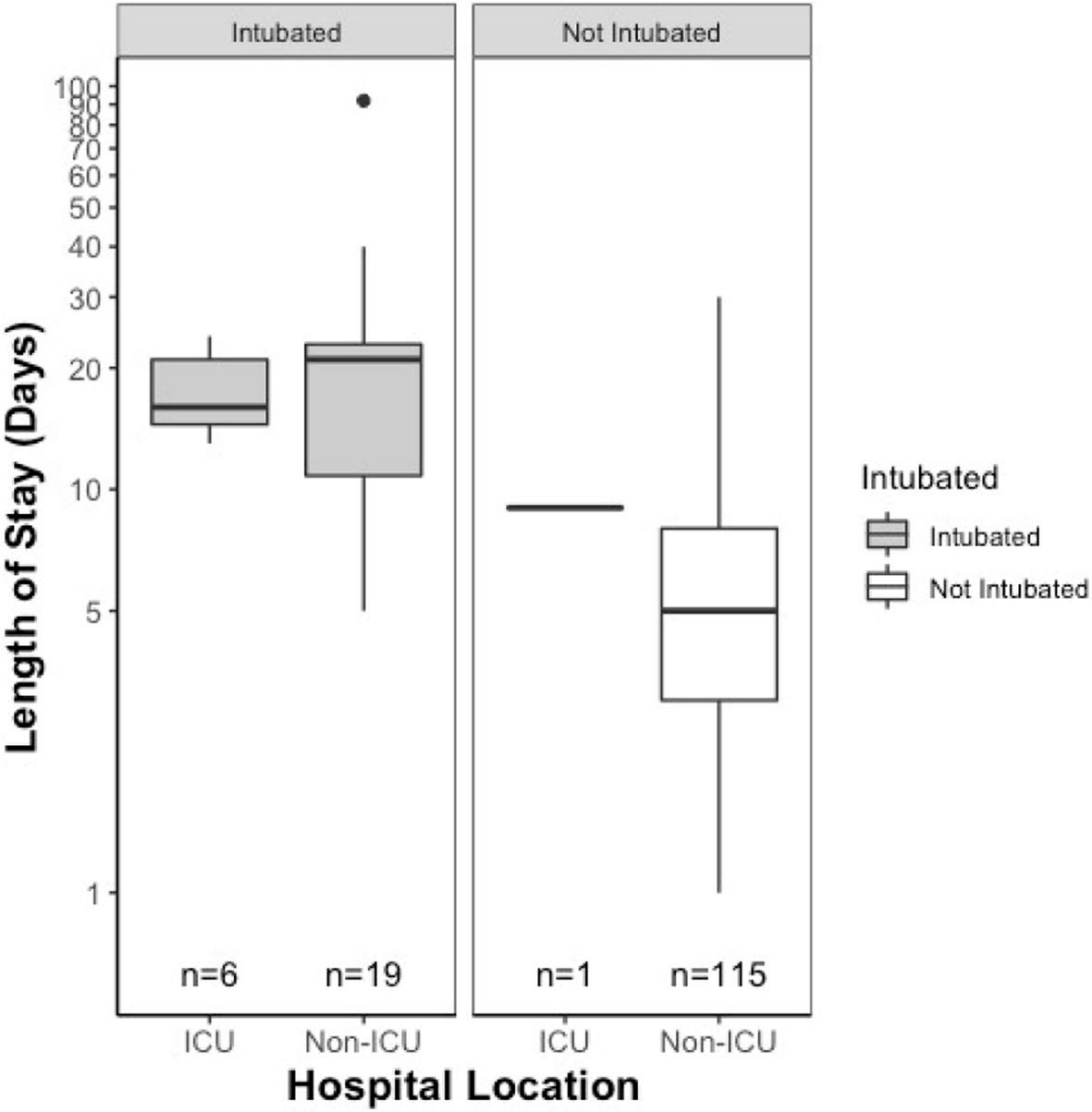
LOS by ICU admission based on intubation status

Non-compliance with maintenance regimen prior to hospitalization was also associated with longer LOS until corrected for multiple comparisons using Bonferroni correction (α=0.05/8=.00625). Because medication non-compliance did not meet the threshold for significance per the Bonferroni correction, there was ultimately no association between any cause of exacerbation and length of hospital stay. ICU admission was associated with longer LOS under univariate analysis (IRR 2.52, *p*<.05) but did not meet threshold for significance under multivariate analysis and thus was not considered to be significant (Figure 5).

Of the five baseline factors measured, being male is a baseline risk factor for increased LOS. Men had a mean LOS of 12.88 days (SD +/- 14.7) as compared to women who had a mean LOS of 6.63 days (SD +/- 5.6). The range for men was 1 to 92 days compared to 1 to 33 for women.

### Factors associated with decreased LOS

Patients with a thymectomy had significantly shorter hospital stays. Mean LOS in those with a thymectomy was 5.81 (SD +/- 6.2) compared to those without a thymectomy who had a mean LOS of 9.53 days (SD +/- 11.0). The range of LOS for events in a patient with a thymectomy was 1 to 36, compared to a range of 1 to 92 for non-thymectomy.

**Fig. 6:**
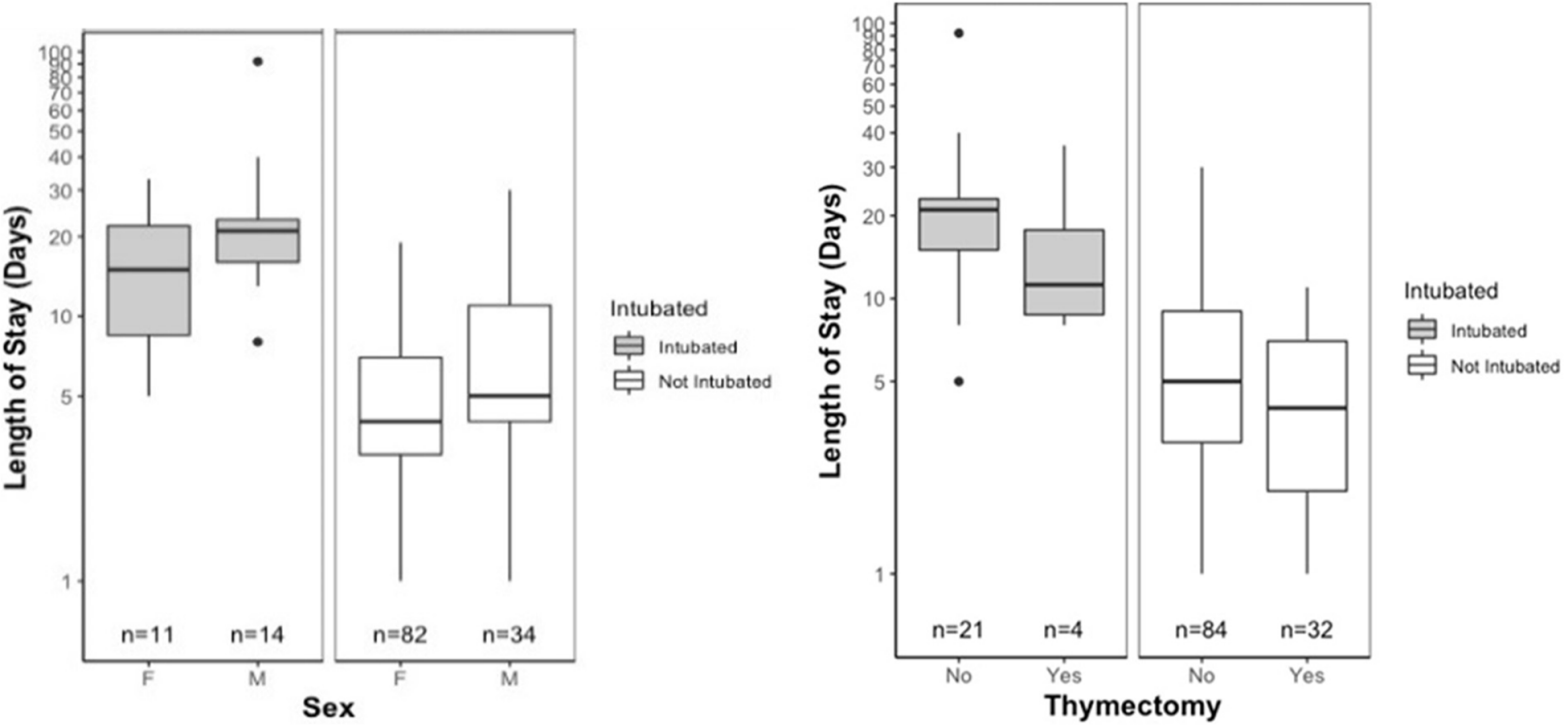
LOS by sex and thymectomy status based on intubation status

Thymectomy status and male sex still had a statistically significant impact on LOS when controlled for by intubation status (Fig. 6). Exacerbations in patients with a thymectomy were more likely to be seropositive, younger, and have more severe disease present at time of initial MG diagnosis compared to patients without thymectomy (Table 4). Plasmapheresis had a statistically significant impact on LOS; however, when controlled for by intubation status, its impact on LOS was in non-intubated patients only. No baseline medications that MG patients were on were found to have an association with length of hospital stay.

**Table 4:** Key clinical features present in the exacerbations in a patient with a thymectomy verses without thymectomy

Clinical Feature	% Of Exacerbations in Patients with Thymectomy (*n* = 36)	% Of Exacerbations in Patients without Thymectomy (*n* = 105)
Male Sex	22%	38%
White Race	67%	99%
Seropositive	75%	62%
Intubated	11%	20%
Average Age	34.7 years	58.5 years
Most Common MGFA Class at Time of Initial Diagnosis	Class 4	Class 2

### Complications

Complications related to treatment developed in 63% of hospital stays (Table 5). Infection was the most common treatment related complication amongst both intubated and non-intubated patients.

**Table 5:** Complications of hospitalization/treatment in intubated and non-intubated patients

Type of Complication	Frequency		Total	Examples of Complications – Medication/Treatment Related
	**Intubated Patients**	**Non-Intubated Patients**		
Cardiovascular	5	18	23	Arrhythmias, Hemodynamic Instability, New Heart Failure, STEMI
Infectious	16	23	39	UTI, HCAP, Bacteremia (from central lines), Sepsis, Cholangitis, Pneumonitis, Tracheitis, Pseudomembranous Colitis, Port Infections, Epidural Abscess, Septic Emboli
Hematological	14	14	28	Deep Vein Thrombosis, Hemorrhage from venous catheter, Thrombocytopenia, Superficial Thrombophlebitis, Anemia, Anaphylactic Shock (plasma product reaction)
Renal	2	5	7	AKI, Electrolyte Disturbance
Respiratory	7	0	7	Failed Extubation Attempts (one or more in a single hospital stay)
Gastrointestinal	2	12	14	Severe Constipation or Diarrhea, Nausea/Vomiting, Pancreatitis, Transaminitis
Musculoskeletal	0	2	2	Severe Myalgia
Neurologic	4	11	15	Delirium, Encephalitis, Cerebral Edema, Acute Ischemic Infarct, New Onset Seizures, Aseptic Meningitis, Optic Neuritis, Narcotic Overdose
**Total**	50	85	135	

*AKI* Acute Kidney Injury, *HCAP* Hospital-Acquired Pneumonia,* STEMI* ST Elevation Myocardial Infarction, *UTI*. Urinary Tract Infection

### Disposition following exacerbation

33% of hospital stays were followed by discharge to a post-acute care facility (Skilled Nursing Facility or Long-Term Acute Care), 7% were discharged home with home health, and one hospital stay resulted in patient death. One patient left AMA. The rest were discharged home.

## Discussion

This study identified use of plasmapheresis, intubation status, and male sex as factors associated with increased LOS in acute MG exacerbation. Thymectomy was associated with shorter LOS. This may relate to the role of the thymus in MG pathogenesis [14]. Intubation was the strongest predictor of hospital LOS. Male sex and thymectomy status still had significant associations with change in LOS when intubation status was accounted for. Plasmapheresis did not.

Plasmapheresis as a contributor to longer hospital stays in non-intubated patients was unsurprising because this hospital’s protocol for treating MG exacerbation with plasmapheresis is one session every other day for a total of five sessions. Therefore, a course of plasmapheresis necessitates a minimum of 10 days in the hospital. The 7.09% of exacerbations treated with both plasmapheresis and IVIg likely represent those with severe refractory exacerbations, which would explain the longer average stay in these patients.

Perhaps the most interesting finding from this data is that patients with a thymectomy spent less time in the hospital for MG exacerbation. Thymectomy has already been shown to be helpful in reducing disease exacerbation and required use of prednisone/immunosuppressants [15]. This could demonstrate another way in which thymectomy helps ameliorate disease burden.

Thymectomy guidelines for MG patients (discussed in further detail below) are largely based on a meta-analysis which examined the benefits of thymectomy as they accrue over time (12 months or longer) [16–18]. Considering that most hospitalizations occurred within the first year of diagnosis, there are at least four possible explanations for why the rate of thymectomies increased with time from diagnosis to hospitalization. It could reflect that MG exacerbations become less frequent over time or that the longer one has had the diagnosis, the more likely they are to have undergone a disease modifying surgery. It could also support prior studies that purport thymectomy reduces frequency of exacerbation [19]. Finally, it may be considered that surgery has the potential to reduce hospital burden even within 12 months. As the hospital burden of MG and its natural history continue to be defined, it would be interesting to see in future studies if there is a relationship between time since surgery and hospital LOS for myasthenia exacerbation, which could indicate that the benefits of thymectomy in MG patients begin sooner than 12 months.

Current guidelines recommend thymectomy in the 10% of MG patients with thymoma [20]. For non-thymomatous patients, thymectomy is recommended in those < 50 years of age without MuSK or LRP4 antibodies but with either generalized MG or refractory disabling ocular MG [15]. Thymectomy may be considered in those 51 to 65 years of age depending on operative risks [16]. For patients 18 to 50 years of age with non-thymomatous generalized AChR antibody-associated MG, thymectomy is based on Grade 1B recommendations [17]. Otherwise, these are Grade 2C recommendations [5]. Beyond these patient populations, decision to proceed with thymectomy is made on an individual basis due to low-quality evidence supporting any benefits. This study includes the populations with weak evidence-based guidelines. Therefore, this finding that patients with thymectomy had shorter hospital stays may serve to help further guide individualized disease management decisions in patient groups where the role of thymectomy, such as those without AChR-Abs and older patients, remains under investigation.

Of these patient groups that remain under investigation, there has recently been more interest in exploring non-AChR serotypes and our findings may be taken in this context. For example, Koing et al. in Journal of Neurology (December 2021) concludes that MuSK-Ab status is associated with a longer need of mechanical ventilation, ICU LOS, and overall hospital-LOS in myasthenic exacerbation compared to AchR-Ab [21]. In contrast, our study did not find an association between seropositivity (including both MuSK and AchR-Ab) and hospital LOS. This could represent a lack of clinical significance in Koing et al.’s findings. Further analysis is still needed to clarify the nuances amongst the different stereotypes.

The study was limited by its retrospective nature. Such studies are prone to misclassification bias. For example, lack of appropriate ICD coding likely excluded potential exacerbations from this dataset. Inadequate documentation likely contributed to the large number of exacerbations with unknown causes and unknown MGFA classes. It is also likely that not all ICU admissions were captured. This is because in the EMR, the location within the hospital reflects the last location before discharge. It is reasonable to predict that if intubation significantly correlated with LOS, so would ICU admission. Given that the rate of intubation was much greater than 5%, there were likely more than 7 ICU admissions which if captured may or may not have changed these results.

In addition to the above limitations inherent to retrospective analyses, these findings may not be generalizable as they were limited to a single academic center and racially homogenous. This may reflect racial demographics of the city of the institution and/or consistency with findings of a retrospective study from 2007 in which Whites were more likely to develop treatment refractory generalized MG [22].

Finally, we did not consider comorbidities as it was beyond the scope of this study primarily assessing the effect of treatment on LOS. Comorbidities are potential confounding variables which may impact LOS as underlying conditions could independently contribute to poor disease control. This is illustrated in a recent retrospective cohort study examining independent risk factors for myasthenic crisis published in Journal of Neuroinflammation (April 2022) concluded that the number of comorbidities, intubation, prolonged mechanical ventilation, and myasthenic crisis triggered by infection were associated with worse outcomes [23]. Similarly, Neumann et al. in a multicenter analysis of myasthenic crisis demanding mechanical ventilation concluded that prolonged ventilation (> 15 days) depended on age, late-onset MG, a high MGFA Class before crisis, pneumonia, resuscitation, and number of comorbidities (> 3 comorbidities) [24]. Of note, it is interesting to contrast this with our results which showed similar average ages at time of initial diagnosis and the same most common MGFA class at time of initial diagnosis in intubated compared to non-intubated patients. Furthermore, our study found no association between age at diagnosis and LOS. This underscores the need for further study of MG disease progression.

Strengths of this paper include its longitudinal nature which spans a decade. It also includes demographics which have generally been excluded from previous MG studies (eg, seronegative, those older than 65 years) and therefore may be generalized to these understudied MG patients as discussed above [19].

Another strength of this study is the statistical model. The conclusions drawn from multivariate analysis are more likely to be accurate than a univariate analysis as it more closely resembles reality where independent and dependent variables rarely occur in isolation from other situational factors.

With a number of novel immunosuppressive drugs under current investigation for generalized MG therapy, this study may help clarify where the gaps are in acute MG treatment. It will be of interest to see if novel therapies have an impact on hospital length of stay for MG exacerbation patients. Continued evaluation of the effect of resources employed during exacerbation management on disease outcomes will be important to establish a basis of comparison as novel treatments advance through clinical trials.

## Supplementary Information

**Additional file 1:** **Supplemental Table 1.** Multi-Variable Incidence Relative Ratio (IRR) for Length of Stay. Adjusted for Multiple Comparison (n=141). *p<0.05. †*p*<.00625 per Bonferroni correction (see statistical methods). **Supplemental Table 2.** Percentage of exacerbations per MGFA class at diagnosis. **Supplemental Table 3.** Percentage of hospitalizations admitted to the general floor, intermediate care (step-down from ICU unit), and ICU.

**Additional file 2:** **Supplemental Figure 1.** Distribution of LOS (days) in hospitalized MG patients with exacerbation. **Supplemental Figure 2.** LOS (days) in intubated patients treated with plasmapheresis and in non-intubated patients treated with plasmapheresis. *N*=141. Circles represent outliers. Bold line represents median. Top and bottom of box represent the 75th and 25th quartile, respectively.

## Data Availability

All data generated or analyzed during this study are included in this published article and its supplementary information files.

## Abbreviations

MG: Myasthenia Gravis
LOS: Length of stay
MUAC-MG: Medications that are to be avoided or be used with caution in patients with MG
VPI: Vaccine preventable infection
VNPI: Vaccine non-preventable infection
EMR: Electronic Medical Records
SD: Standard Deviation
PEG: Percutaneous Endoscopic Gastrostomy
IRR: Incidence Relative Ratio
STEMI: ST-Elevated Myocardial Infarction
UTI: Urinary Tract Infection
AKI: Acute Kidney Injury
HCAP: Health Care Associated Pneumonia
