# Supplementary material for: Analysis of length of stay and treatment emergent complications in hospitalized myasthenia gravis patients with exacerbation
Source: BMC Neurol. 2023 Jan 12;23:12. doi: 10.1186/s12883-022-02922-9 (PMC9835387; doi:10.1186/s12883-022-02922-9)
Supplement: Supplementary file 2 — Additional file 2: Supplemental Figure 1. Distribution of LOS (days) in hospitalized MG patients with exacerbation. Supplemental Figure 2. LOS (days) in intubated patients treated with plasmapheresis and in non-intubated patients treated with plasmapheresis. N=141. Circles represent outliers. Bold line represents median. Top and bottom of box represent the 75th and 25th quartile, respectively. [file 12883_2022_2922_MOESM2_ESM.docx]

**Supplemental Data:**

**Supplemental Figure 1. Distribution of LOS (days) in hospitalized MG patients with exacerbation**

**Supplemental Figure 2. LOS (days) in intubated patients treated with plasmapheresis and in non-intubated patients treated with plasmapheresis**

**
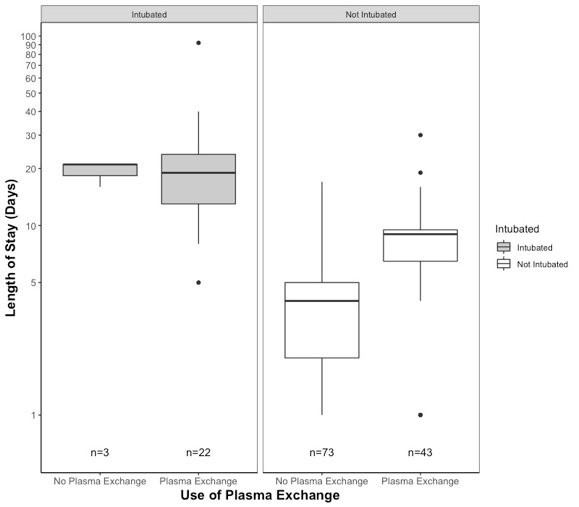
**

*N=141. Circles represent outliers. Bold line represents median. Top and bottom of box represent the 75th and 25th quartile, respectively.*

**Supplemental Table 1. Multi-Variable Incidence Relative Ratio (IRR) for Length of Stay**

| **Parameter** | **IRR ( 95% CI)** | ***p* Value** |
| --- | --- | --- |
| Age at diagnosis | 1.01 (1.00-1.01) | .10 |
| Sex (Male) | 1.26 (1.02-1.55) | .028^*^ |
| Thymectomy (Yes) | 0.67 (0.50-0.91) | .01^*^ |
| Antibody Status (Positive) | 1.19 (0.93-1.52) | .16 |
| Unknown Antibody Status | 1.60 (0.99-2.59) | .05 |
| **MGFA Status at Diagnosis** |  |  |
| MGFA Class I | 1.00 | -- |
| MGFA Class II | 0.86 (0.63-1.16) | .31 |
| MGFA Class III | 1.07 (0.78-1.48) | .67 |
| MGFA Class IV | 0.65 (0.44-0.95) | .027 |
| MGFA Class V | 1.18 (0.80-1.74) | .40 |
| Unknown MGFA Status | 0.94 (0.67-1.33) | .73 |
| **Cause of Exacerbation** |  |  |
| Unknown | 1.00 | -- |
| Diagnostic | 1.41 (0.96-2.06) | .08 |
| Medication-Related | 1.09 (0.81-1.47) | .56 |
| Non-Compliance | 1.55 (1.12-2.25) | .007 |
| Procedural | 1.16 (0.72-1.86) | .55 |
| VNPI | 1.17 (0.85-1.61) | .32 |
| VPI | 1.04 (0.74-1.45) | .84 |
| Other | 1.29 (0.90-1.85) | .16 |
| **Hospital Stay Location** |  |  |
| General | 1.00 | -- |
| ICU | 0.71 (0.48-1.06) | .09 |
| Intermediate Care | 1.15 (0.87-1.50) | .32 |
| **Use of Ventilation** |  |  |
| Intubated | 2.10 (1.60-2.74) | < .001^*^ |
| Non-Invasive Ventilation | 1.03 (0.84-1.26) | .79 |
| **Medications Used to Treat Exacerbation** |  |  |
| IVIG | 0.95 (0.76-1.20) | .66 |
| Plasmapheresis | 2.13 (1.67-2.71) | < .001^*^ |

*Adjusted for Multiple Comparison (n=141). *p<0.05. †p<.00625 per Bonferroni correction (see statistical methods)*

**Supplemental Table 2. Percentage of exacerbations per MGFA class at diagnosis.**

| **MGFA Class at Diagnosis** | **Number (%)**  **(n=141)** |
| --- | --- |
| MGFA Class I | 17% |
| MGFA Class II | 30% |
| MGFA Class III | 20% |
| MGFA Class IV | 11% |
| MGFA Class V | 6% |

**Supplemental Table 3. Percentage of hospitalizations admitted to the general floor, intermediate care (step-down from ICU unit), and ICU.**

| **Hospital Stay Location** | **Number (%)**  **(n=141)** |
| --- | --- |
| General | 81.8% |
| Intermediate Care | 13.5% |
| ICU | 5% |
